# Tunable terahertz biosensing and shielding using a graphene-enhanced dielectric resonator absorber

**DOI:** 10.1038/s41598-025-08982-4

**Published:** 2025-07-20

**Authors:** Patri Upender, Kuldeep Yadav

**Affiliations:** School of Electronics Engineering, Vellore Institute of Technology, Chennai, Tamil Nadu India

**Keywords:** Absorber, Biosensor, Wideband, Refractive index, Graphene, Biophysics, Cancer, Engineering, Electrical and electronic engineering

## Abstract

In this paper, we present a compact terahertz (THz) absorber based on a graphene-enhanced dielectric resonator structure, designed to achieve both wideband and narrowband absorption. The proposed absorber demonstrates ultra-wideband absorption from 1.07 to 13.3 THz, achieving a bandwidth of 12.23 THz, along with a sharp narrowband peak at 6.81 THz under specific tuning conditions. Tunability is achieved by adjusting the chemical potential (µc) of graphene, while the structure remains polarization-insensitive and maintains high absorption stability up to incident angles of 70°. Simulation results validated through an equivalent circuit model demonstrate the absorber’s strong performance with a maximum sensitivity (S) of 5.11 THz/RIU, figure of merit (FOM) of 601.2 RIU^−1^, and a quality factor (Q) of 1322.5, making it highly effective for biosensing applications, including the detection of chemicals, viruses, and cancerous cells. Additionally, for EM interference shielding, the absorber exhibits an absorption effectiveness exceeding 10 dB, and a reflectance effectiveness within 1 dB, confirming its high efficiency in suppressing reflected waves. These results highlight the absorber’s potential for versatile applications in stealth, shielding, and practical biosensing.

## Introduction

In recent years, the THz frequency range, has attracted significant interest for its potential applications across a range of fields, including biomedical sensing, security, and telecommunications^1–4^. Due to its unique position in the electromagnetic (EM) spectrum, lying between the microwave and infrared bands, THz waves exhibit both non-ionizing properties and the ability to penetrate various materials such as plastics, fabrics, and biological tissues, making them especially valuable in non-destructive testing and imaging^5,6^. Moreover, THz frequency holds immense potential for applications in both advanced communication systems and sensitive biosensing technologies. However, the inherent susceptibility of THz frequencies to EM interference also makes EM shielding essential to ensure signal clarity, device integrity, and measurement accuracy in these applications. EM shielding has become increasingly important due to the pervasive nature of EM interference in modern technology^7–9^. As our dependence on electronic devices and wireless communication grows, so does the risk of EM interference, which can degrade device performance, affect communication reliability, and even compromise sensitive biomedical instruments. Effective EM shielding solutions are thus crucial for protecting electronic systems, ensuring secure data transmission, and maintaining operational stability across various sectors. Biosensing is also advancing as a vital tool in medical diagnostics and environmental monitoring. The need for rapid, accurate, and non-invasive detection of biomolecules, pathogens, and chemicals drives the development of high-performance biosensors, which benefit significantly from tailored EM absorbers^10–15^. Absorbers used in biosensing applications effectively localize EM waves, enhance sensor S, and improve the signal-to-noise ratio. Importantly, EM absorbers are engineered with wideband and narrowband absorption characteristics to meet these application-specific requirements.

Few researchers in^16–20^ explored various advancements in electromagnetic energy absorption, microwave power reception, and wireless power transmission, including innovative meta-surface arrays, hybrid designs, phase gradient techniques, and applications in microwave hyperthermia technology. In addition to these, works in^21–23^ discuss further advancements in absorption enhancement, including the use of monolayer graphene with a multiple-resonator approach for ultra-broadband near-infrared absorption, nanoscale-thick ITO hemispherical shells as saturable absorbers for Q-switched lasers, and the modulation of single-layer graphene’s narrow-band absorption through surface plasmon polaritons in the near-infrared region. These advancements in material design and properties, especially involving metamaterials, are central to progress in EM shielding and biosensing due to their customizability and unique EM characteristics arising from engineered structures. Unlike conventional materials, metamaterials can be designed to exhibit unusual behaviours, such as negative refractive indices, strong localization of EM fields, and near-perfect absorption at tailored frequencies^24–27^. These properties make metamaterials particularly effective for applications where traditional materials fall short, as they enable enhanced control over EM waves and increase the absorber’s efficiency. In recent years, metamaterials have been used to design ultra-thin, flexible absorbers that are not only effective across a wide range of frequencies but also compact, making them ideal for integration into sensitive biosensing devices and wearable technologies. Among various types of metamaterials, dielectric resonator-based metamaterials are emerging as a powerful solution for EM shielding and biosensing applications^28,29^. Dielectric resonators exhibit low loss at high frequencies, such as the THz range, and can be precisely engineered to achieve both narrow and wideband absorption profiles. Their high permittivity and ability to concentrate EM fields make them particularly suitable for biosensing applications, where they enhance the interaction between the sensor and the target analyte, leading to improved detection S. Furthermore, dielectric resonator-based metamaterials can be integrated with other components, such as graphene layers or conductive materials, to optimize the absorption properties further, ensuring high efficiency in both wideband and narrowband regimes. Furthermore, integrating the graphene layer in the proposed absorber plays a pivotal role in enhancing absorption performance. Due to its unique electrical and optical properties, graphene enables tunable absorption through its chemical potential, providing a degree of flexibility that traditional materials lack^30^. This tunability allows the proposed absorber to achieve both wideband and dual-band absorption characteristics, making it adaptable for precise applications such as biosensing and EM shielding. Additionally, graphene’s high conductivity and low relaxation time enable it to absorb THz waves effectively, reducing reflection and increasing absorption efficiency. The inclusion of graphene in the absorber design not only improves overall S but also enhances the FOM and Q, making it ideal for high-performance THz sensing and shielding applications.

Researchers have extensively studied advanced materials to develop optimized THz absorbers with versatile features^31–41^. For instance, authors in^37^ have designed an ultra-narrow band dielectric-based refractive index sensor that exhibits an absorption rate larger than 0.99 with full width at half-maximum (FWHM) of 0.38 nm, high S of 1052 nm/RIU and a large FOM of 2768. In another instance^38^, demonstrates an ultra-wideband THz absorber using dielectric circular truncated cones, offering broadband absorption from 0.34 to over 10 THz. Further, the dual ultra-narrowband dielectric metamaterial absorber in^39^, constructed from an asymmetric silicon dimer array on a silica substrate, achieves an extremely low FWHM of 0.008 nm and a high-Q, making it highly suitable for precise sensing applications. Additionally, in^36^, researchers proposed an ultrathin silicon ring-based design for tunable THz biosensing, which achieves peak sensitivities of 0.20 and 0.10 THz per RIU. Another innovative design, combining VO₂ and graphene, presented in^40^, delivers both broadband and selective absorption in conductive and insulative states, respectively. The application of THz metamaterials (MMs) for sensing avian influenza virus subtypes H1N1, H5N2, and H9N2 is highlighted in^42^, reaching a peak S of 0.54 THz/RIU. The graphene-based absorber in^41^, incorporating a sinusoidal-patterned dielectric resonator, demonstrates a wide bandwidth of 6.58 THz. Additionally, split-ring resonator configurations have been proposed in THz MMs^11,12,43^, offering designs with electromagnetically induced transparency-like Fano resonance features. While these absorbers exhibit excellent performance in specific areas, they still face certain challenges, including limitations in bandwidth, structural bulkiness, reliance on metallic elements, and the inability to provide simultaneous tunability, narrow, and wideband capabilities in a single design.

Achieving both wideband and narrowband absorption within a single absorber has become essential for multifunctional applications, such as electromagnetic (EM) shielding and biosensing. However, existing refractive index (RI) sensors often focus either on broadband or narrowband functionality, rarely achieving both simultaneously within a compact and tunable platform. In this work, we present a novel dielectric resonator absorber enhanced with graphene, capable of simultaneous ultra-wideband and narrowband absorption, thus overcoming this limitation. Such dual functionality allows for flexible operation across varied frequency ranges, enabling comprehensive coverage for shielding against EM interference while also providing the spectral precision needed for biosensing at specific THz frequencies. This dual capability not only improves the efficiency of the absorber but also reduces the need for multiple specialized devices, simplifying design and deployment across applications. In EM shielding, wideband absorption helps suppress a broad range of interfering frequencies, enhancing device performance in secure communication or sensitive measurement environments. Additionally, in stealth applications, broadband absorbers help reduce the radar cross-section (RCS) across a wide frequency span, improving concealment and operational efficiency in dynamic environments. Meanwhile, the narrowband response within the same absorber is critical for biosensing, as it enables precise detection of biological analytes by focusing on specific frequencies where these analytes exhibit distinct resonance. The proposed absorber incorporates a compact, dielectric-based metamaterial design, achieving exceptionally wideband absorption from 1.07 to 13.3 THz while maintaining a tunable narrowband peak at 6.81 THz. This tunability is facilitated by modulating the chemical potential (µc) of graphene, allowing dynamic adjustment of the absorption profile in real time, which is particularly advantageous for adaptive sensing applications.

In addition to its dual absorption performance, the absorber is polarization-insensitive and maintains absorption levels above 80% for oblique incident angles up to 70°, making it suitable for practical deployment. The device’s functionality is further verified through an equivalent circuit model and comprehensive simulations, which demonstrate high sensitivity (S), a superior quality factor (Q), and a high figure of merit (FOM). These features make the proposed absorber especially effective for detecting cancerous cells and various viruses, addressing a key challenge in THz biosensing with improved robustness, tunability, and multifunctionality.

## Proposed design

The proposed absorber comprises multiple functional layers. The bottom gold (Au) layer, with a conductivity of $\:\sigma\:=4.5\times\:{10}^{7}S/m$, and a periodicity of $\:{W}_{d}\times\:{L}_{d}=80\times\:80\:\mu\:m$, acts as a reflective surface to maximize absorption with a thickness of 0.1 μm. A polyimide substrate, with a permittivity of $\:{\epsilon\:}_{s}=3.5$, and a thickness of t_p_ = 1 μm, is placed on top of the Au layer to provide dielectric support. Polyimide was selected due to its superior properties in the terahertz (THz) frequency range, particularly its low dielectric constant and low loss tangent, which help minimize signal attenuation and improve absorber efficiency. Additionally, polyimide offers excellent mechanical flexibility, thermal stability, and chemical resistance, making it highly suitable for flexible and conformal THz devices, which is especially important in practical biosensing and stealth applications. Above the substrate, a graphene (Gr) layer is introduced with a chemical potential (µ_c_) of 0.1 eV, a relaxation time (τ) of 0.1 ps, and a temperature of T = 300 K with a thickness of 0.34 nm. This graphene layer allows for tunable absorption due to its unique electronic properties. On top of the graphene layer, a silicon (Si)-based circular ring dielectric resonator (SCDR), with a permittivity of 11.9, a thickness of t_si_ = 1.5 μm, and inner and outer ring radii of r_in_ = 30 μm and r_o_ or r_out_ = 50 μm, respectively, is positioned on top to enhance resonant absorption at desired frequencies. The SCDR serves as the primary absorber due to its high permittivity, which supports strong EM resonance and wideband absorption. Its low dielectric loss minimizes energy dissipation, while its compatibility with microfabrication ensures precise implementation. On the other hand, the graphene layer introduces tunability through its adjustable chemical potential, enabling control over the absorption frequency and bandwidth. Its strong interaction with THz waves enhances absorption efficiency, and its ultrathin structure ensures a compact and lightweight design.

**Fig. 1:**
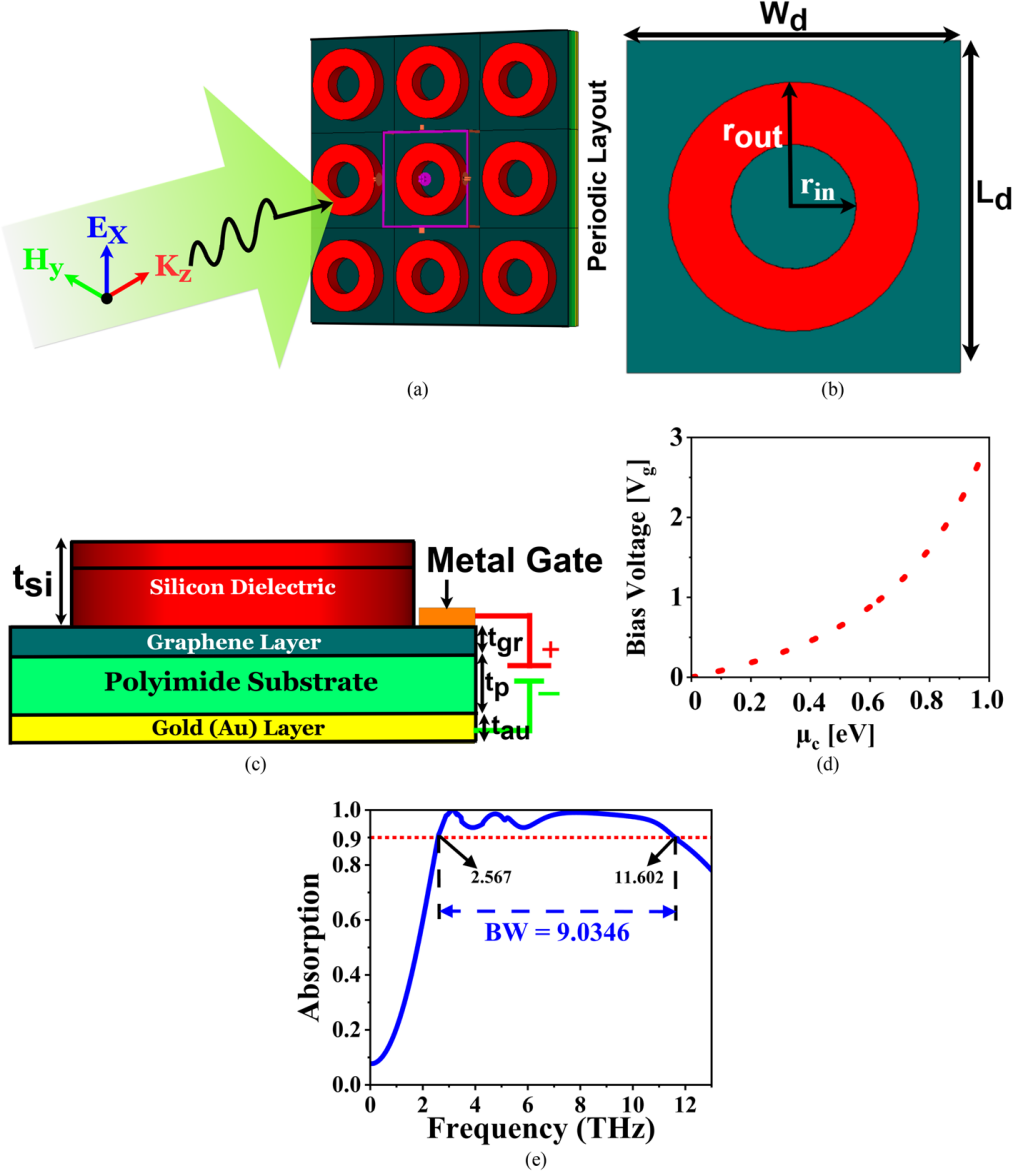
Proposed absorber: (**a**) Periodic layout, (**b**) Unit cell, (**c**) Front view, (**d**) V_g_ vs. µ_c_, and (**e**) Absorption characteristics.

The periodic layout with the incident EM wave and the unit cell dimensions is depicted in Fig. 1a, b, respectively. The simulation and optimization of the proposed absorber were carried out using CST Microwave Studio 2021. The boundary conditions were configured as unit cell boundaries along the x- and y-axes, while an open (add space) boundary condition was applied along the z-axis. An electric field, polarized along the x-direction, was incident on the absorber along the z-axis. A frequency-domain solver was employed for the analysis, utilizing a mesh setup with 126,812 tetrahedral cells to ensure high accuracy in capturing the absorber’s performance. The front view of the proposed absorber is shown in Fig. 1c. Figure 1d presents the variation in the chemical potential (µ_c_) of graphene as a function of the applied DC bias voltage (V_g_). The plot highlights that µ_c_ can be effectively tuned from 0 eV to 1 eV by controlling the electrostatic bias. In thinner graphene films, smaller voltage changes are sufficient to achieve this tuning range. In contrast, thicker graphene layers require comparatively higher voltages to produce similar shifts in µ_c_. This tunability enables dynamic control over the electrical conductivity of graphene, which in turn influences the absorber’s performance characteristics. The absorption characteristics of the absorber are illustrated in Fig. 1e. As shown in the figure, the absorber demonstrates wideband absorption, ranging from 2.567 THz to 11.602 THz, with a bandwidth of 9.0346 THz, and a centre frequency (f_c_) of 7.0845 THz.

This wideband absorption is achieved due to the combination of the Gr layer and the SCDR. The Gr layer allows for dynamic tuning of absorption by controlling its chemical potential, while the resonator helps confine the EM waves, leading to high absorption efficiency across the wide frequency range. Here, graphene is modelled as a two-dimensional surface with its properties defined by the surface conductivity based on the Kubo formula. Its EM behaviour is represented using surface conductivity derived from the Kubo formula, ensuring accurate modelling in the terahertz regime.

The surface conductivity of graphene is given by the following Eqs.^41,44–48^.

1 $${\sigma _S}\left( {\omega ,\tau ,{\mu _c},T} \right)={\sigma _{IB}}\left( {\omega ,\tau ,{\mu _c},T} \right)+{\sigma _{EB}}\left( {\omega ,\tau ,{\mu _c},T} \right)$$

2 $${\sigma _{IB}}\left( {\omega ,\tau ,{\mu _c},T} \right) \approx - j\frac{{{e^2}{k_B}T}}{{\pi {h^2}\left( {\omega - j{\tau ^{ - 1}}} \right)}}\left[ {\frac{{{\mu _c}}}{{{k_B}T}}+2\ln \left[ {\exp \left( { - \frac{{{\mu _c}}}{{{k_B}T}}} \right)+1} \right]} \right]$$

3 $${\sigma _{EB}}\left( {\omega ,\tau ,{\mu _c},T} \right) \approx - j\frac{{{e^2}}}{{4\pi \hbar }}\ln \left[ {\frac{{2\left| {{\mu _c}} \right|+\left( {\omega - j{\tau ^{ - 1}}} \right)\hbar }}{{2\left| {{\mu _c}} \right| - \left( {\omega - j{\tau ^{ - 1}}} \right)\hbar }}} \right]$$

4 $$\:{V}_{g}=\frac{te{\mu\:}_{c}^{2}}{\pi\: \hbar {v}_{f}^{2}{\epsilon\:}_{0}{\epsilon\:}_{r}}+{V}_{0}$$

where σ_IB_ and σ_EB_ are intra and interband conductivities, ω is the angular frequency, τ is the relaxation time, charge of the electron (e), Boltzmann constant (*k*_*B*_), Planck constant (*ħ*), $\:{V}_{g}$ is the gate voltage, t (layer thickness), ε_0_​ (permittivity of free space), and ε_r_​ (relative permittivity of the medium) and $\:{V}_{0}$ is the voltage compensation dependent on the chemical doping, t.

The σ_IB_ component of σ_s_ remains active within the examined frequency range and could be adjusted utilizing µ_c_​ in response to the employed DC potential, according to the expression specified in Eq. (4). Thus, graphene can enable tuning of the absorber’s characteristics. The absorption A(ω) is typically derived from the reflection R(ω), and transmission T(ω) of the absorber, where ω is the angular frequency. Since the transmission is generally zero for a perfect absorber due to the metallic backing (Au), absorption can be expressed as:

5 $${\text{A(}}\omega {\text{) = 1}} - {\text{R(}}\omega {\text{) }}$$

Here, R(ω) can be computed based on the reflection coefficient at the material interface. The dimensions of the proposed absorber are chosen as follows: the unit cell periodicity is $\:1.89\times\:{\lambda\:}_{centre}$ ​, the polyimide substrate thickness is $\:0.0236\times\:{\lambda\:}_{centre}$​, the silicon resonator thickness is $\:0.0354\times\:{\lambda\:}_{centre}$ ​, the inner ring radius is $\:0.709\times\:{\lambda\:}_{centre}$​, and the outer ring radius is $\:1.18\times\:{\lambda\:}_{center}$ ​, where $\:{\lambda\:}_{centre}$ corresponds to the wavelength at the centre frequency of 7.0845 THz.

This combination of materials and design not only broadens the absorption bandwidth but also ensures a strong response over the entire frequency range, making the absorber highly suitable for THz applications.

## Progression of the proposed absorber

**Fig. 2:**
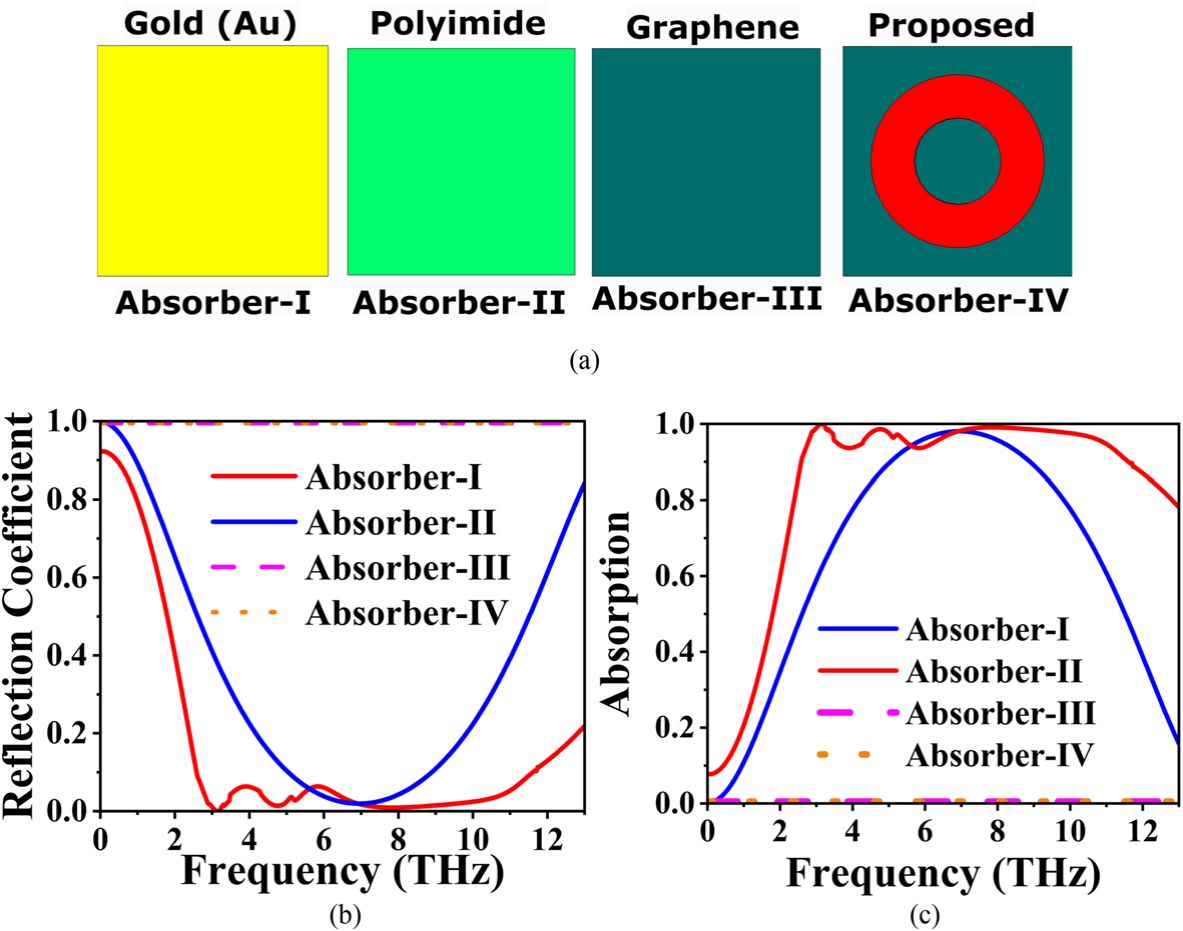
(**a**) Development of the proposed absorber, (**b**) Reflection Coefficient plot, and (**c**) Absorption plot.

The development of the proposed absorber, along with its reflection and absorption characteristics, is illustrated in Fig. 2. The first configuration, designated as absorber-I, comprises the Au layer, which exhibits zero absorption and functions as a perfect reflector across the measured frequency range. This behaviour is attributed to the high conductivity of the Au layer, which reflects incident EM waves rather than absorbing them. In the second configuration, referred to as absorber-II, the polyimide layer also demonstrates zero absorption, further contributing to the reflective properties of the structure. The polyimide serves primarily as a dielectric spacer, supporting the overall architecture without significantly affecting the absorption characteristics. Absorber-III, which consists of the Gr layer, displays significant absorption behaviour, operating effectively within the frequency range of 5.0469 THz to 8.0959 THz, achieving absorption levels exceeding 90%. This remarkable performance can be attributed to the tunable electrical properties of graphene, which allow for effective modulation of the EM waves. The interaction between the Gr layer and the EM fields enhances the resonant absorption due to its inherent conductive characteristics and adjustable µ_c_. Finally, the proposed absorber, incorporating an SCDR, achieves wideband absorption, as depicted in the reflection and absorption plots. The design synergistically combines the contributions of the graphene layer and the resonator, resulting in a broad absorption spectrum. This makes the proposed absorber particularly advantageous for applications requiring high S and performance across a wide range of frequencies.

## Equivalent circuit

**Fig. 3:**
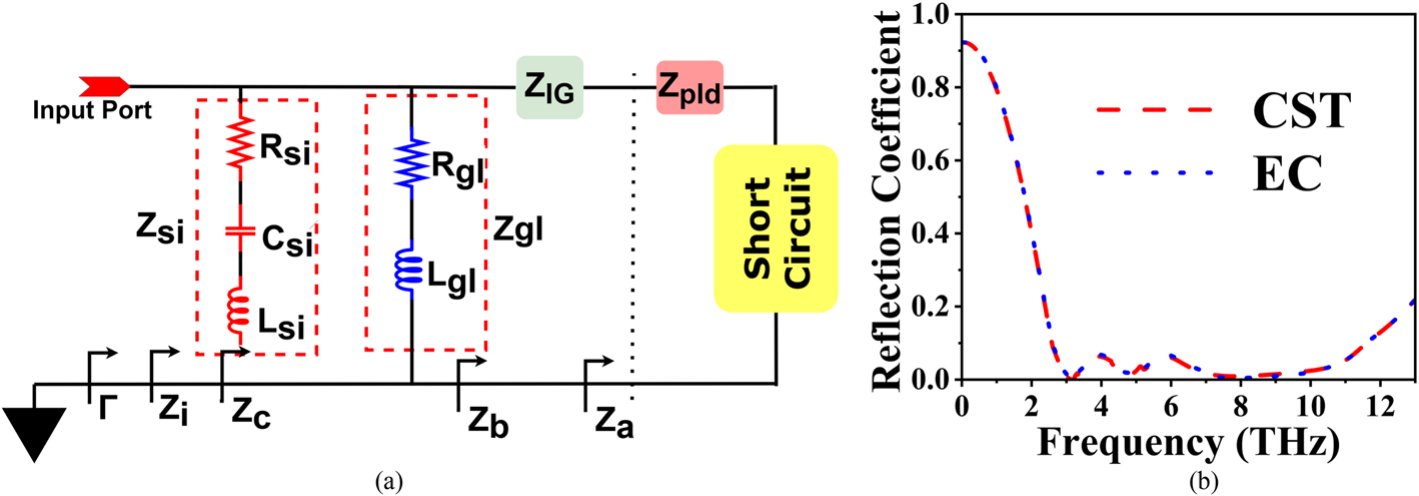
(**a**) EC (C_si_=0.02 fF, L_si_=0.025 nH, R_si_=27.23 Ω, R_gl_=150Ω, and L_gl_=0.005 nH) and (**b**) Reflection Cofficient plot.

The equivalent circuit (EC) of the proposed absorber is designed to capture the interactions between its different layers, representing their EM behaviour as shown in Fig. 3. Starting from the bottom, the gold (Au) layer is modeled as a short circuit, providing a termination point with zero impedance. Above this, the polyimide substrate and ion gel layer are represented by series impedances Z_pid_, and Z_ig_, respectively, accounting for the inductive and capacitive effects of these layers. They influence wave propagation and contribute to phase matching. In parallel with the combined impedance of the polyimide and ion gel layers, the graphene layer is represented as a combination of a resistor R_gl_ and an inductor L_gl_​, capturing graphene’s resistive losses and inductive behaviour, contributing to absorption over a wide frequency range due to its tunable surface conductivity. Further, the silicon-based circular dielectric resonator (SCDR) is modelled as a parallel RLC circuit, with resistance Rsi​, inductance Lsi ​, and capacitance Csi ​, collectively represented by the impedance Zsi​. This accounts for the resonator’s energy storage and dissipation characteristics. The impedance provided by the polyimide substrate is given by:

6 $$\:{Z}_{pid}=j\omega\:{L}_{pid}+\frac{1}{j\omega\:{C}_{pid}}$$

Here L_pid_, and C_pid_ are the inductance and capacitance associated with the polyimide substrate, and ω = 2πf is the angular frequency. Next, the impedance provided by the ion gel layer is given by:

7 $$\:{Z}_{ig}=j\omega\:{L}_{ig}+\frac{1}{j\omega\:{C}_{ig}}$$

Similar to the polyimide layer, the ion gel has its inductive and capacitive components.

For graphene, since it has resistance R_gl_ and inductance L_gl_​, its impedance is:

8 $$\:{Z}_{gl}={R}_{gl}+j\omega\:{L}_{gl}$$

The impedance of the SDCR is modelled as an RLC circuit and is given by:

9 $$\:{Z}_{Si}={\left(\frac{1}{{R}_{si}}+j\omega\:{C}_{si}+\frac{1}{j\omega\:{L}_{si}}\right)}^{-1}$$

Here, R_si_​, L_si_​, and C_si_ ​ are the resistance, inductance, and capacitance of the SCDR.

The total impedance Z_i_ is calculated as:

10 $$\:{Z}_{i}={\left(\frac{1}{{Z}_{pid}+{Z}_{ig}}+\frac{1}{{Z}_{gl}}+\frac{1}{{Z}_{si}}\right)}^{-1}$$

Finally, the reflection coefficient (Γ) is calculated by:

11 $$\:{\Gamma\:}=\frac{{Z}_{i}-{Z}_{0}}{{Z}_{i}+{Z}_{0}}$$

Where Z_0_​ is the characteristic impedance of free space.

Figure 3b shows the reflection coefficient plots obtained from both CST Microwave Studio simulations and the EC model. The comparison reveals excellent agreement between the two, indicating that the equivalent circuit accurately captures the EM behaviour of the proposed absorber. This consistency validates the accuracy of the equivalent circuit model in replicating the key absorption characteristics, making it a reliable tool for predicting the absorber’s performance across the frequency range.

## Parametric analysis

**Fig. 4:**
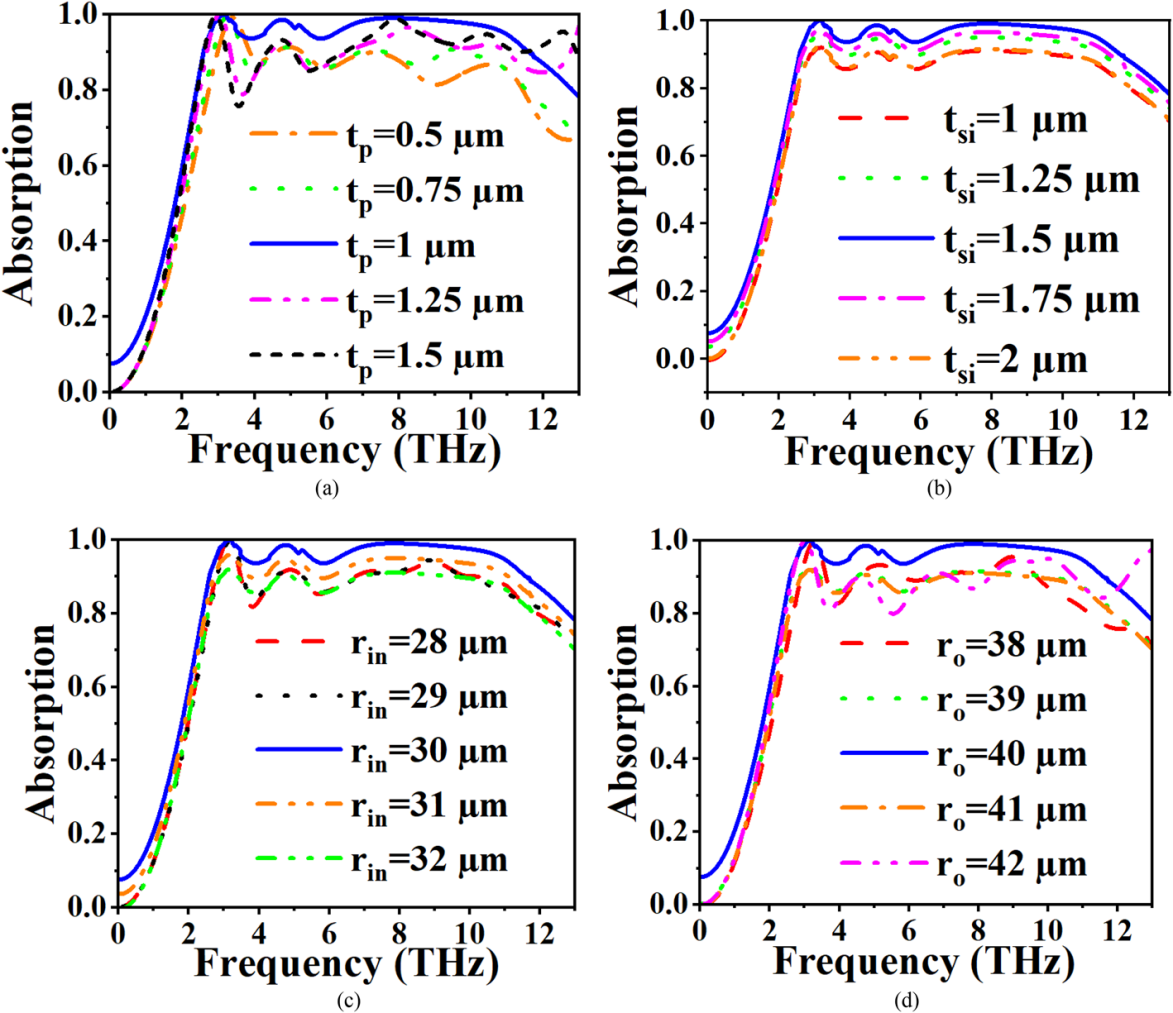
(**a**) Development of the proposed absorber, (**b**) Reflection Coefficient plot, and (**c**) Absorption plot.

The parametric analysis is conducted on the physical parameters to investigate their influence on the absorber’s performance. The key physical parameters considered in this analysis include the substrate thickness t_p_​, the DR thickness t_si_​, the inner ring radius r_in_​, and the outer ring radius r_out_​, respectively as depicted in Fig. 4. In the parametric analysis, only one parameter is varied at a time while keeping the others constant, to isolate and understand the effects of each parameter on the absorber’s performance. First, the substrate thickness t_p_​ is varied from 0.5 μm to 1.5 μm in steps of 0.25 μm, as shown in Fig. 4a. It is observed that when t_p_=1, the absorber exhibits a wideband absorption with optimal performance. As t_p_ increases beyond 1 μm, a slight decrease in absorption is noticed, which can be attributed to the increased separation between the graphene layer and the dielectric resonator, affecting the coupling between the resonator modes and the incident EM waves. For values of t_p_ ​less than 1 μm, a more pronounced decrease in absorption occurs due to insufficient dielectric spacing, which disrupts the resonance conditions necessary for wideband absorption. Next, the dielectric resonator (DR) thickness t_si_ is varied from 1 μm to 2 μm, as depicted in Fig. 4b. At t_si_=1.5 μm, the absorber demonstrates excellent absorption characteristics, with over 90% absorption across the band. For values of t_si_ ​greater than 1.5 μm, the absorber still exhibits wideband absorption, but the absorption efficiency decreases slightly. This can be explained by the fact that an increase in DR thickness modifies the effective refractive index and impedance matching, which, while maintaining broad absorption, leads to a shift in resonant frequencies. Conversely, for t_si_ ​values less than 1.5 μm, the absorption drops to around 80%, as the thinner resonator is less capable of effectively trapping and dissipating the incident energy.

Similarly, the inner radius of the circular ring resonator r_in_​ is varied from 28 μm to 32 μm, as shown in Fig. 4c. When r_in_=30 μm, the absorber achieves wideband absorption. For other values, especially as r_in_ increases, a gradual deterioration in absorption is observed. This behaviour can be attributed to the geometric tuning of the resonator, where deviations from the optimal r_in_ affect the resonant frequencies and reduce the overall absorption efficiency by altering the resonant mode distributions within the dielectric resonator.

Finally, the outer radius r_out_ is analyzed, with variations from 38 μm to 42 μm as shown in Fig. 4. For r_out_=40 μm, the absorber again displays wideband absorption. However, for values of r_out_ ​ either greater or less than 40 μm, the absorption begins to decrease. The outer radius strongly influences the mode confinement within the resonator, when r_out_ deviates from the optimal value, the absorber’s impedance matching to free space worsens, leading to less effective energy coupling and, consequently, reduced absorption efficiency. Thus, the parametric analysis underscores the importance of precise tuning of the physical parameters for achieving optimal wideband absorption. The identified optimal values for each parameter ensure the highest absorption efficiency and widest bandwidth, making the absorber well-suited for applications in the THz regime.

## Stability analysis

**Fig. 5:**
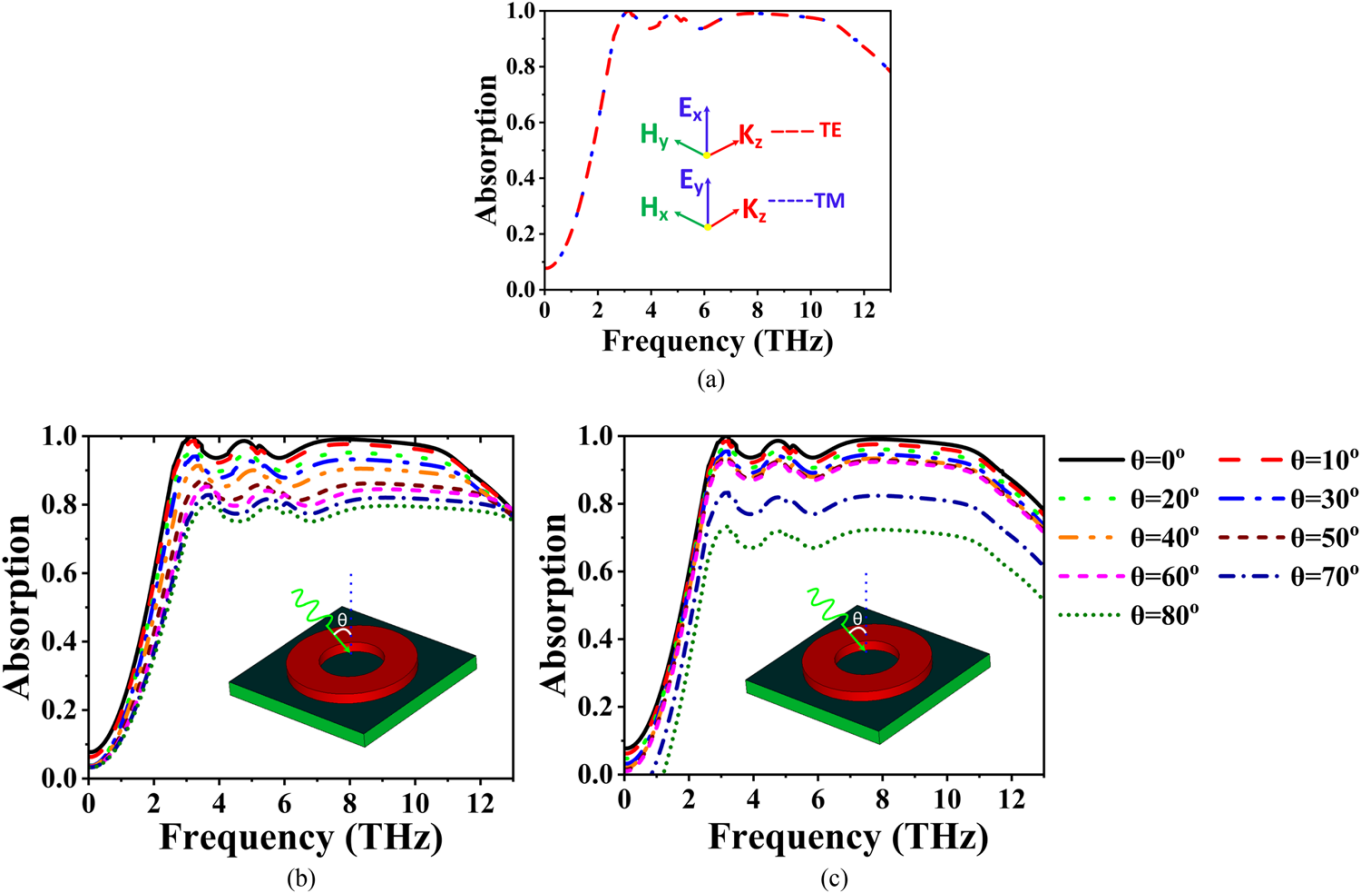
Absorption responses of the proposed absorber regarding (**a**) polarization angle variations and (**b**) TE mode, as well as (**c**) TM mode for different angles of incidence (θ).

The stability of the proposed absorber is verified under both TE (Transverse Electric) and TM (Transverse Magnetic) wave incidences, as depicted in Fig. 5. In Fig. 5a, it is evident that the absorber maintains similar absorption performance for both TE and TM incidences through a series of polarization angles. This consistent behaviour is primarily due to the symmetric design of the proposed structure, which ensures polarization insensitivity. Subsequently, the absorber’s performance is analyzed for different incidence angles (θ) under TM incidence, as shown in Fig. 5b. The results demonstrate that the absorber achieves more than 80% absorption for incidence angles θ < 70°. However, at θ = 80°, the absorption slightly decreases to around 70%. Similarly, under TE incidence, the absorber exhibits more than 80% absorption for angles θ < 70°, but at θ = 80°, the absorption reduces further to approximately 60%. This reduction at higher angles is expected due to the increased reflection and reduced interaction of the incident waves with the absorber’s surface at steeper incidence angles. The stability of the proposed absorber is verified for TE and TM incidence as illustrated in Fig. 5. Figure 5a, shows the proposed absorber exhibits similar absorption performance for both TE and TM incidence across various polarization angles.

This is because of the symmetry of the proposed structure. Next, the absorber performance is investigated for different θ under TM incidence. The absorber exhibits more than 80% of absorption up to θ < 70°. But for θ = 80°, the absorber shows absorption of around 70%. Similarly, the absorber exhibits more than 80% of absorption up to θ < 70^o^. But for θ = 80°, the absorber shows absorption of around 60%.

## Field distribution

**Fig. 6:**
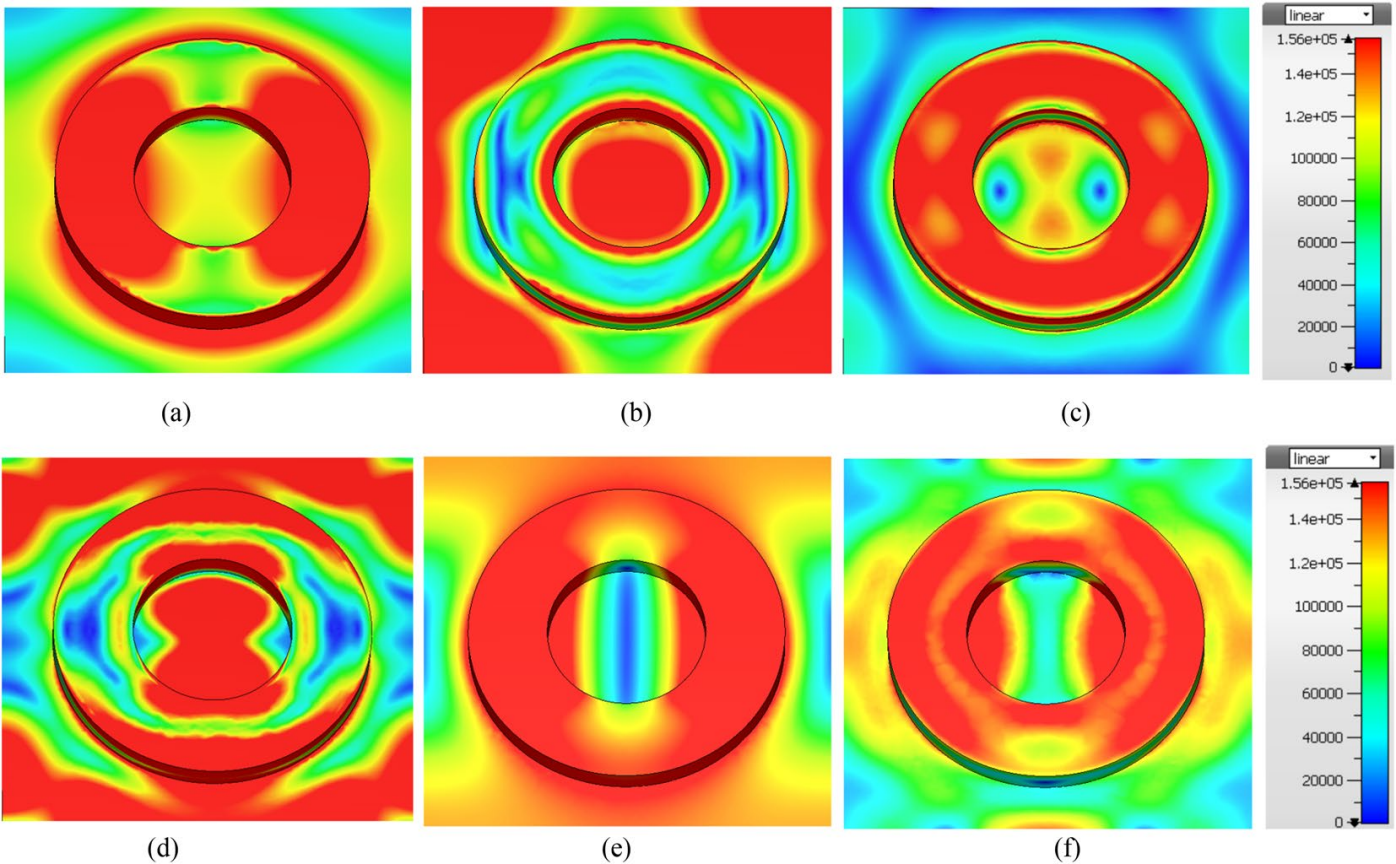
EF distribution on the absorber at: (**a**) 3.16 THz, (**b**) 4.7 THz, (**c**) 6.032 THz, (**d**) 8.05 THz, (**e**) 9.41 THz, and (**f**) 11.38 THz.

The electric field (EF) distribution on the proposed absorber at different frequencies is depicted in Fig. 6. At 3.16 THz, the absorption is primarily due to the silicon-based circular dielectric resonator (SCDR), as the strong EF intensity is concentrated on the DR, with a small concentration also observed on the graphene layer. This leads to excellent absorption at this frequency, as shown in Fig. 6a. At 4.7 THz, the absorber exhibits slightly reduced absorption, corresponding to a lower EF concentration on the DR, as depicted in Fig. 6b. At 6.032 THz, the absorber demonstrates a higher EF concentration on the DR, contributing to increased absorption at this frequency, as shown in Fig. 6c. This can be attributed to the resonance of the DR at this frequency. Similarly, at 8.05 THz, as shown in Fig. 6d, the field distribution is primarily concentrated on both the DR and the graphene layer, resulting in near-perfect absorption at this frequency. At 9.41 THz, as illustrated in Fig. 6e, the EF distribution is again focused on the DR, leading to strong absorption. Finally, at 11.38 THz, as depicted in Fig. 6f, the EF distribution is primarily located on the DR, with slightly less concentration on the circular ring, corresponding to a slight reduction in absorption. Moreover, the field distribution penetrates the silicon and clusters both at the edges and inside the material due to resonant coupling and the high permittivity of silicon. At the resonant frequencies, the THz waves interact strongly with the silicon dielectric resonator, causing energy concentration inside the material. This penetration is a result of the high permittivity contrast between silicon and the surrounding medium, as well as the mode structure formed by the interaction between the THz waves and the silicon, enhancing absorption and tuning capabilities. This analysis highlights that the absorption is predominantly driven by the SCDR, with enhanced absorption achieved through the combined interaction of the graphene layer and the SCDR, resulting in near-perfect absorption across the wideband.

## EM shielding performance

The proposed absorber is analyzed for its EM shielding performance, specifically targeting wideband absorption that is crucial for applications in EM interference shielding and stealth technologies. The absorber’s excellent absorption capabilities make it highly effective in attenuating EM waves, thus meeting the stringent demands of shielding performance. The effectiveness of the shielding is evaluated using standard equations, which quantify the contribution of different mechanisms absorption, reflection, and multiple reflections to the overall shielding effectiveness^49,50^.

The total shielding effectiveness (E) is given by the equation:

6 $$\:E={E}_{A}+{E}_{R}+{E}_{M}$$

7 $$\:{E}_{A}=-10\times\:{\text{log}}_{10}T/1-R$$

8 $$\:{E}_{R}=-10\times\:{\text{log}}_{10}1/R$$

9 $$\:{E}_{M}=-10\times\:{\text{log}}_{10}\left(1-{R}^{2}\right)$$

Here, E_A_ is absorption effectiveness, E_R_​ is the reflectance effectiveness, E_M_​ accounts for the effectiveness of multiple reflections, T represents transmittance, and R is the reflectance of the absorber.

**Fig. 7:**
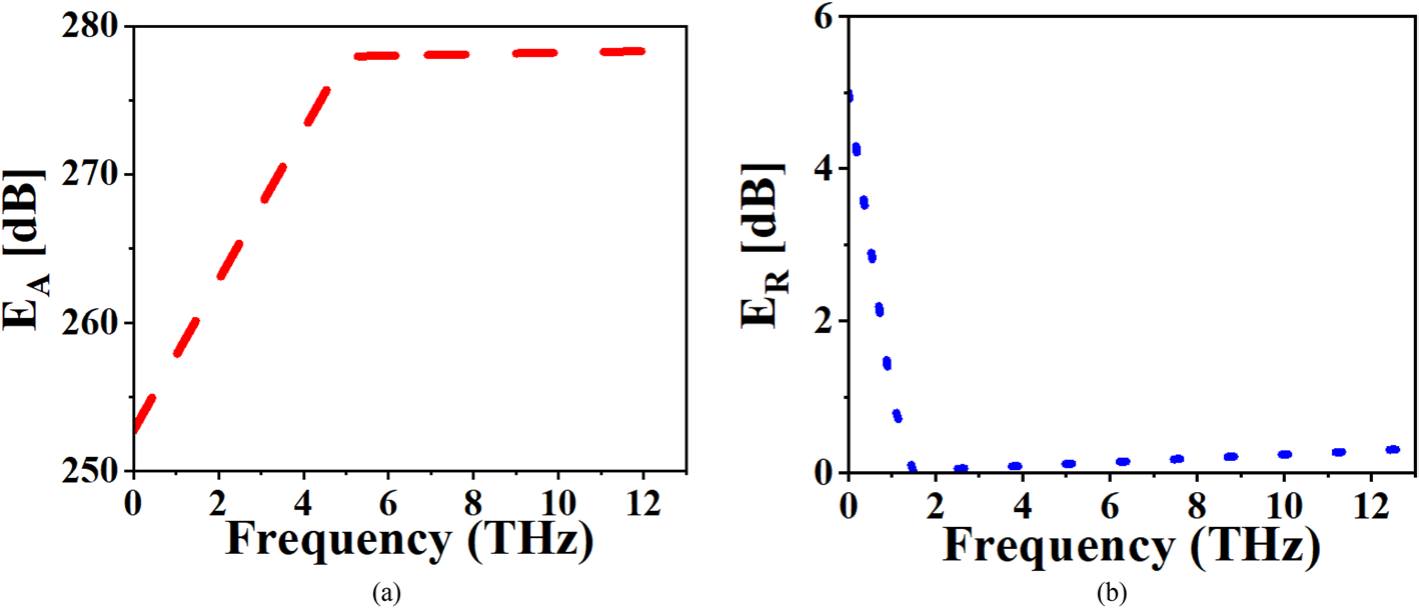
EM shielding Performance.

A practical criterion for effective EM shielding requires the E_A_​ to exceed 10 dB, ensuring that E_M_​ have minimal influence on the overall performance. As shown in Fig. 7a, the proposed absorber exhibits an absorption effectiveness E_A_​ significantly higher than 10 dB, confirming its strong absorption capabilities. Moreover, the reflectance effectiveness E_R_ ​ remains within 1 dB, indicating that the absorber is designed to minimize the reflection of EM waves, efficiently directing most of the incident wave energy into absorption as shown in Fig. 7b. This demonstrates that the proposed absorber not only excels in absorbing incident EM energy but also effectively suppresses reflection and multiple reflections, making it an excellent candidate for EM interference shielding and stealth applications in the THz regime.

## Graphene chemical potential

**Fig. 8:**
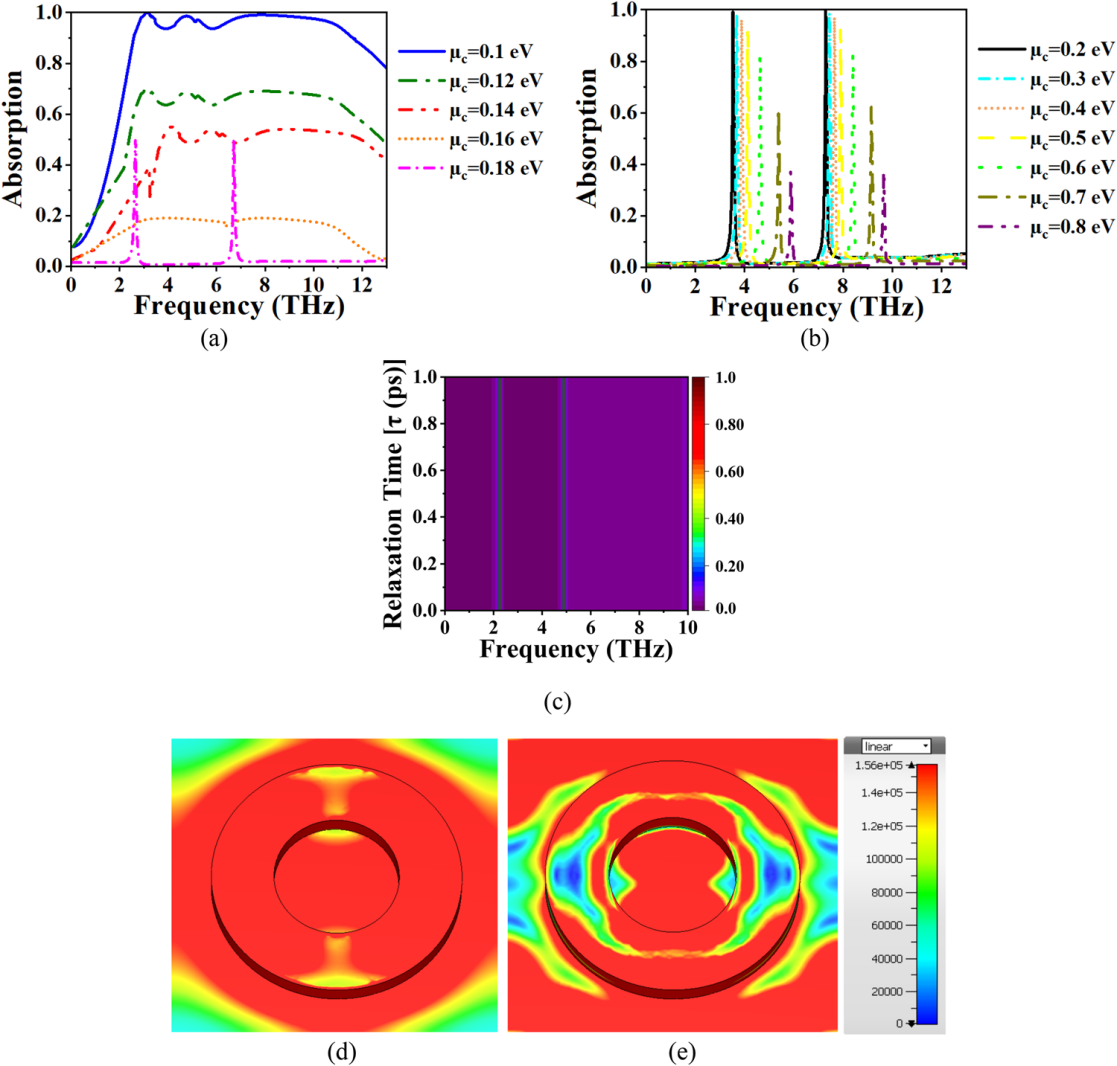
(**a**), (**b**) Absorption vs. µ_c,_ (**c**) τ vs. frequency. EF distribution at (**d**) 3.52 THz, and (**e**) 7.29 THz.

The absorber’s performance is evaluated by varying the chemical potential µ_c_​ of the graphene layer from 0.1 to 0.8 eV as depicted in Fig. 8a, and Fig. 8b. Graphene’s surface conductivity σ(µ_c_) is a function of both the chemical potential and frequency. As µ_c_ increases, the conductivity of the graphene layer changes, impacting the resonant behaviour of the absorber. At lower µ_c_ (0.2 eV), graphene exhibits strong resonance characteristics, leading to efficient EM wave absorption and the formation of well-defined dual-band absorption peaks as depicted in Fig. 8b. At µ_c_ = 0.2 eV, the graphene layer exhibits optimal conductivity for resonating with both frequency bands (7.2993 THz and 3.523 THz). This resonance enhances the energy dissipation in the graphene, leading to near-perfect absorption (99.9973% and 99.988%). The resonances are determined by the interplay between the electric field concentration and the impedance matching of the structure. As µ_c_ ​ increases beyond 0.2 eV, the surface conductivity of graphene shifts, which modifies the impedance matching between the graphene layer and the incident EM waves. This change causes a deterioration in absorption performance because the resonant conditions required for optimal absorption are altered. Despite this, the structure still produces dual-band behaviour for µ_c_ ​ values up to 0.6 eV, though the absorption efficiency gradually drops.

This is because, at higher µ_c_​, the graphene transitions to a regime where it becomes more reflective and less absorptive. For µ_c_ > 0.6 eV, the conductivity of graphene becomes less favourable for absorption, and the material starts reflecting more of the incident EM energy, leading to a further decrease in absorption. This is typical in graphene-based absorbers, where higher µ_c_ ​ values shift the resonance conditions away from the desired operating frequencies, reducing the energy dissipation and thus the absorption performance. The tunable behaviour of graphene by varying µ_c_ ​ is particularly advantageous for biosensing applications. By adjusting µ_c_, the resonant frequencies can be shifted to target-specific molecular signatures, providing a way to fine-tune the absorption spectrum for specific sensing tasks. Figure 8c presents the variation in the absorber’s resonant frequencies as a function of graphene’s relaxation time (τ) at a fixed chemical potential of µ_c_ = 0.2 eV. The results demonstrate that the resonant peaks remain nearly unchanged across different τ values, indicating that the relaxation time does not significantly impact the resonance positions or absorption strength in this design. This confirms that the tunability of the device is primarily governed by changes in the µ_c_, rather than the scattering rate of charge carriers in graphene. Hence, the absorber offers stable operation even under realistic variations in graphene quality, which is beneficial for practical device integration.

The dual-band absorption observed in the range of µ_c_ = 0.2 eV to 0.5 eV offers high S, which is ideal for detecting biochemical changes in the THz frequency range. The corresponding EF distributions at µ_c_ = 0.2 eV are shown in Fig. 8d, e. At 3.52 THz, the EF is predominantly localized on the outer perimeter of the dielectric resonator, with strong vertical confinement and field enhancement near the resonator–graphene interface. This intense field concentration supports efficient light–matter interaction, which is crucial for increasing S. At 7.29 THz, the EF distribution becomes even more complex and dynamic, as shown in Fig. 8e, with the field highly concentrated on both the inner and outer ring edges of the resonator and penetrating deeply into the structure. This results in a strong coupling between the dielectric and graphene layers, enhancing energy dissipation in the graphene and yielding near-perfect absorption. Quantitatively, the peak field magnitudes reach approximately 1.5 × 10⁵ V/m, and these high-field regions act as localized “hot spots,” amplifying the absorber’s responsiveness to even minimal refractive index variations. Such field localization increases the effective interaction volume, enabling the detection of extremely small biochemical changes.

## Proposed absorber as biosensor

**Fig. 9:**
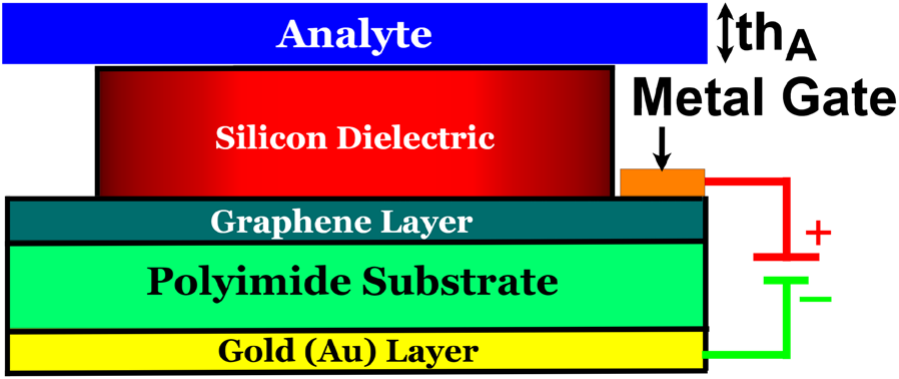
Proposed absorber structure with overlaid analyte.

**Fig. 10:**
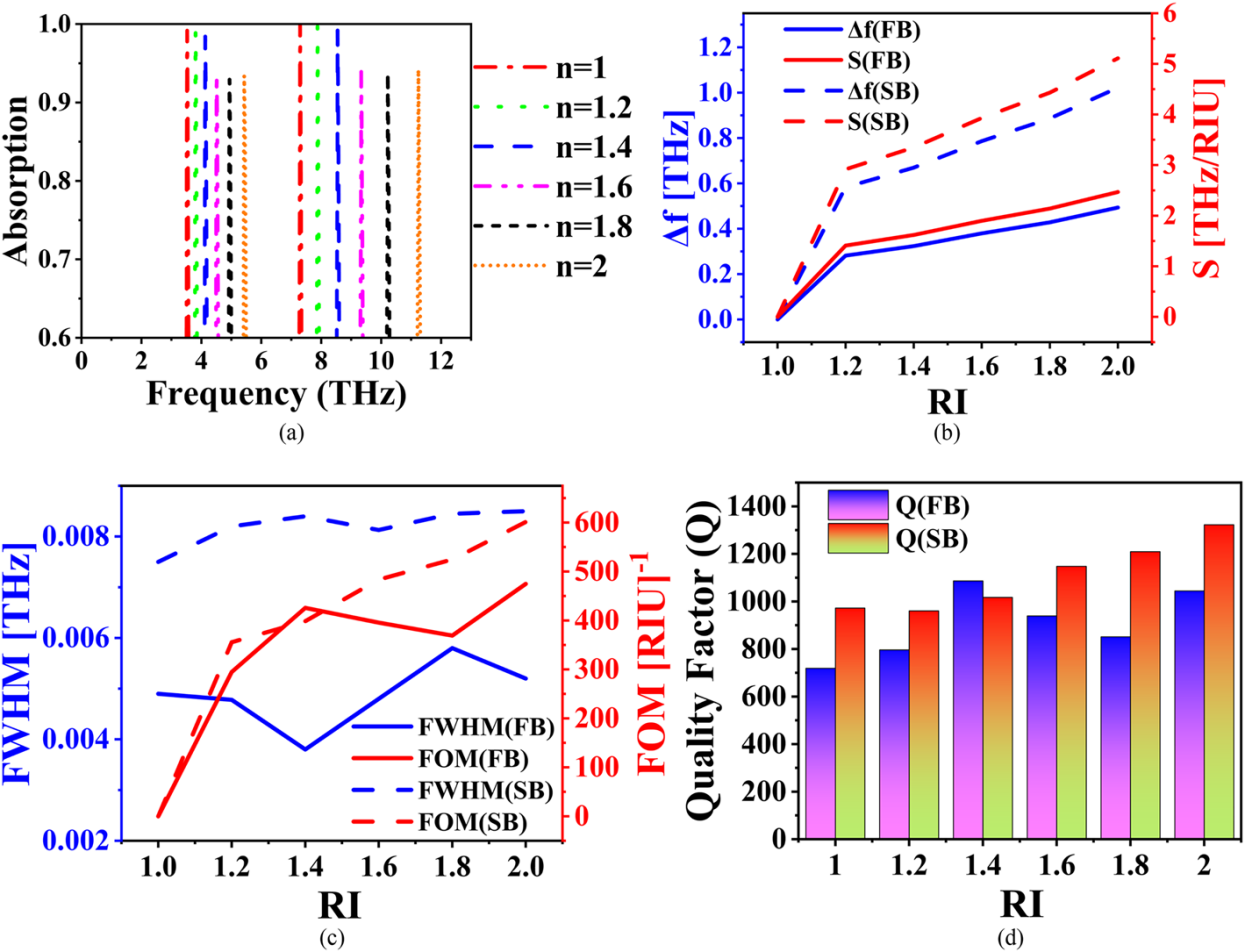
Sensing performance: (**a**) absorption response for various values of n, (**b**) RI vs. Δf, S, (**c**) RI vs. FWHM, FOM, and (**d**) RI vs. Q.

The investigation reveals that at µ_c_ = 0.2 eV, the proposed absorber exhibits dual-band response with resonances at 3.5253 THz and 7.2993 THz, achieving absorption rates of 99.9973% and 99.988%, respectively. This dual-band characteristic marks the absorber highly apt for biosensing purposes, capable of detecting various chemicals and viruses. Figure 9 illustrates the proposed absorber (µ_c_ = 0.2 eV) with an analyte layer on top, with thickness th_A_, and depicts the biasing mechanism, including a metal gate. Figure 10a presents the absorption characteristics as the refractive index (RI or n) of the analyte varies from *n* = 1 to *n* = 2, in steps of 0.2. The corresponding plots of the change in resonant frequency (Δf) and S for different RI values are shown in Fig. 10b. The frequency shift Δf is calculated as the difference between the current and previous resonance frequencies (f_p_), while S is determined using $\:S=\varDelta\:f/\varDelta\:n$​, where Δn represents the change in refractive index. At RI = 2, the maximum S of 0.4937 THz/RIU (in the first band (FB)) and 1.022 THz/RIU (in the second band (SB)) are observed, indicating the absorber’s exceptional capability for biosensing due to its high S to changes in the analyte’s RI. Additionally, Fig. 10c depicts the FWHM and the FOM as a function of RI. FOM is calculated as $\:FOM=S/FWHM$​, and a maximum FOM of 474.71 RIU^−1^ (FB) and 601.27 RIU^− 1^ (SB) is achieved for RI = 2. Moreover, the Q ($\:Q={f}_{p}/FWHM$​​) reaches maximum values of 1044.5 and 1322.6 for the FB and SB, respectively, as shown in Fig. 10d. These high values of *S*, *FOM*, and Q underscore the exceptional performance of the proposed absorber, making it a highly effective and precise platform for biosensing applications. The absorber’s ability to detect even subtle changes in the refractive index of the analyte, along with its strong resonance behaviour, ensures a high degree of accuracy and reliability in identifying various chemicals or biological substances.

Next, the proposed sensor is analyzed for its ability to detect various chemicals, including ethanol (*n* = 1.36), water (*n* = 1.333), glycerine (*n* = 1.47), sodium chloride (*n* = 1.54), ethylene glycol (*n* = 1.41), carbon disulfide (*n* = 1.63) and benzene (*n* = 1.5)^28,51^. Table 1 presents the sensor’s performance metrics for detecting these chemicals. The results demonstrate that the sensor exhibits excellent characteristics for S, FOM, and Q. These high-performance indicators make the sensor an outstanding choice for detecting these chemicals with precision and efficiency, further solidifying its potential for practical applications in chemical detection and environmental monitoring. The proposed sensor is also tested for detecting different viruses^52–54^, like malaria in different stages (n_1_ = 1.373 and n_2_ = 1.383), dengue (*n* = 1.4), influenza A (*n* = 1.48), healthy skin (*n* = 1.844), and cancerous cells (*n* = 2.0149) as shown in Table 2. The sensor successfully detects malaria (n_1_​) with S values of 1.60 THz/RIU and 3.18 THz/RIU in the FB and SB, respectively. For dengue detection, the sensor indicates high S of 1.513 THz/RIU in FB and 3.131 THz/RIU in SB. While detecting influenza A, the sensor achieves a maximum S of 1.527 THz/RIU and 3.307 THz/RIU in FB and SB, respectively.

**Table 1:** Sensing performance for various chemicals

Name	RI	Δf (THz)		S (THz/RIU)		FWHM (THz)		FOM (RIU^− 1^)		Q	
		FB	SB	FB	SB	FB	SB	FB	SB	FB	SB
Water	1.333	0.4904	0.9447	1.472	2.836	0.0045	0.0078	327.2606	363.7099	892	1056.154
Ethanol	1.36	0.5784	1.1327	1.606	3.146	0.00473	0.0084	339.6758	374.5701	867.2304	1003.095
Ethylene Glycol	1.41	0.6144	1.2688	1.498	3.094	0.00381	0.0081	393.3167	382.0536	1086.089	1057.049
Glycerine	1.47	0.7334	1.5807	1.560	3.363	0.00473	0.00808	329.8997	416.2366	900	1098.267
Benzene	1.5	0.7974	1.6457	1.594	3.291	0.0044	0.00855	362.4545	384.9591	982.0455	1045.497
NaCl	1.54	0.8344	1.8977	1.545	3.514	0.00451	0.00899	342.6131	390.9076	966.2971	1022.358
Carbon disulphide	1.63	1.0474	2.1717	1.662	3.447	0.00487	0.00805	341.3839	428.2165	938.6037	1175.776

Additionally, the sensor effectively differentiates between healthy skin cells and cancerous cells, offering very high S of 1.836 THz/RIU in FB and 3.781 THz/RIU in SB. These results demonstrate the sensor’s excellent performance in detecting various viruses, highlighting its effectiveness and precision across a range of biological applications.

**Table 2:** Sensing performance for various chemicals.

Virus	RI	Δf (THz)		S = Δf/Δn (THz/RIU)		FWHM (THz)		FOM (RIU^− 1^)		Q	
		FB	SB	FB	SB	FB	SB	FB	SB	FB	SB
Malaria (n1)	1.373	0.5974	1.186	1.60160	3.18150	0.0052	0.007	308.001	0.010329	792.5	1115.789
Malaria (n2)	1.383	0.6014	1.248	1.57023	3.26031	0.0048	0.007	324.428	0.010049	852.272	1170.13
Dengue	1.4	0.6054	1.252	1.5135	3.13175	0.0053	0.008	285.028	0.010988	777.589	1042.19
Influenza A	1.48	0.7334	1.587	1.52791	3.30770	0.0058	0.008	261.629	0.012643	728.938	1032.67
Skin (Healthy)	1.844	1.4634	2.936	1.73388	3.47950	0.0043	0.007	400.435	0.008689	1151.73	1294.93
Cancerous	2.049	1.926	3.966	1.83679	3.78141	0.0051	0.008	360.156	0.010499	1068.70	1390.12

It is vital to describe the fabrication process of the proposed absorber. The proposed absorber is designed to be compatible with well-established fabrication techniques. The process begins with depositing a thin gold layer (0.1 μm) onto a carrier substrate, which serves as a reflective ground plane. A polyimide film (1 μm thick) is then spin-coated on top and thermally cured. Polyimide was chosen for its low dielectric loss, flexibility, and stability in the terahertz range, making it ideal for applications like biosensing and stealth technologies.

Next, a monolayer of graphene is transferred onto the polyimide using a wet-transfer process. The graphene layer allows for tunable absorption through electrostatic biasing, thanks to its adjustable chemical potential. Finally, a silicon-based circular dielectric resonator is patterned above the graphene using standard lithography and etching techniques. This resonator, with a thickness of 1.5 μm and ring radii of 30 μm and 50 μm, is responsible for enhancing the absorption at specific THz frequencies. This layer-by-layer process is straightforward and aligns with common cleanroom fabrication methods, making the proposed absorber both practical and scalable.

Table 3 provides a comprehensive comparison between the performance of the proposed absorber and other designs reported in recent literature. The proposed sensor demonstrates notably high S, indicating its capability to precisely detect even minimal changes in the RI of an analyte. This high S makes the sensor exceptionally suitable for applications requiring accurate RI detection, such as biochemical and environmental sensing. Additionally, the enhanced S, combined with the high Q and FOM, further confirms the sensor’s effectiveness and reliability in various sensing applications. Moreover, the proposed sensor is polarization-insensitive (PI) and exhibits excellent tunability (TU) through chemical potential modulation of graphene, enabling flexible and dynamic control over its sensing performance under varying operating conditions.

**Table 3:** Comparative analysis of the proposed absorber with other recent studies

Refs.	NB	f_*r*_ (THz)		S (THz/RIU)		FOM (RIU^− 1^)		Q		T (µm)	PI	TU
		FB	SB	FB	SB	FB	SB	FB	SB			
^31^	2	1.42	2.99	0.28	1.48	1.2	24.6	7.11	59.8	10.8	Yes	No
^32^	2	1.8	2.26	0.1875	0.36	7.2	19.1	120	94	8.6	Yes	No
^33^	2	1.77	2.45	-	1.9	-	229	6.91	296	4.8	No	No
^34^	2	0.76	1.28	0.47	0.51	9.4	14.4	23	3.7	5.4	Yes	No
^35^	2	4.88	10.9	1.35	2.95	4.67	8.8	15.25	29.88	2.52	Yes	Yes
^36^	2	4.515	4.91	0.2	0.1	4.76	3.5	105.2	211.84	3.65	Yes	Yes
^53^	2	4.3	7.44	0.57	1.15	1.14	1.55	8.17	9.73	–	Yes	Yes
This work	2	3.52	7.29	2.468	5.11	474.7	601.2	1044.3	1322.5	2.5	Yes	Yes

PI: Polarisation Insensitivity, TU: Tunability.

## Conclusion

The proposed THz absorber, based on a graphene-integrated dielectric resonator, successfully combines ultra-wideband and tunable narrowband absorption in a compact design. It maintains high stability across wide incident angles and achieves significant performance metrics: a maximum sensitivity of 5.11 THz/RIU, a figure of merit of 601.2 RIU⁻¹, and a quality factor of 1322.5, demonstrating its effectiveness in detecting biochemical analytes with high precision. For electromagnetic shielding, the absorber achieves an absorption effectiveness greater than 10 dB and maintains reflectance effectiveness below 1 dB, ensuring minimal EM wave reflection and optimal absorption. These results underscore the absorber’s suitability for dual-purpose applications in biosensing and EM interference mitigation, setting a strong benchmark for future tunable THz absorber designs.

## Data Availability

Data availability: The datasets generated and/or analyzed during the current study are not publicly available due to the sensitive and proprietary nature of aspects of the research, which are related to ongoing projects, the data that support the findings of this study are available upon reasonable request from the corresponding author.
